# Strategies to Improve the Performance of Li Metal Anode for Rechargeable Batteries

**DOI:** 10.3389/fchem.2020.00409

**Published:** 2020-05-08

**Authors:** Zhongliang Hu, Jingying Li, Xiaojing Zhang, Yirong Zhu

**Affiliations:** Department of Inorganic Nonmetallic Material, College of Metallurgy and Material Engineering, Hunan University of Technology, Zhuzhou, China

**Keywords:** Li metal anode, Li metal batteries, Li dendrite, solid electrolyte interphase, solid-state electrolyte, structured anode

## Abstract

Li metal batteries have been considered as the most promising batteries with high energy density for cutting-edge electronic devices such as electric vehicles, autonomous aircrafts, and smart grids. However, Li metal anode faces the issues of safety and capacity deterioration, which are closely related to Li dendrite growth. In this paper, we review the main strategies to improve the performance of Li metal anode. Due to Li dendrite's catastrophic influence, suppression of Li dendrite growth is prerequisite for each strategy. Apart from Li dendrite, interfacial resistance between electrolyte and electrode, ionic conductivity of electrolytes, mechanical strength, and volume fluctuation of Li metal anode are also discussed in these strategies. We outline these strategies based on the classifications of constructing solid electrolyte interphase, engineering of solid-state electrolyte and adopting matrix for Li metal anode. Each strategy is illustrated and discussed in detail by exemplification. For better understanding, some important theories related to Li metal anode have been also introduced. Finally, the outlooks for future research of Li metal anode are presented.

## Introduction

Li-ion batteries (LIBs) have achieved great success since their commercial application in portable electronic devices in 1991 (Dunn et al., 2011; Goodenough and Park, 2013), but they have an inherent deficiency of low energy density, which can not meet the growing demand in the large scale application of cutting-edge electronic devices such as electric vehicles, autonomous aircrafts, and smart grids. Therefore, other battery chemistries beyond Li-ion need to be developed (Hong et al., 2019; Zhang Y. et al., 2019; Yang et al., 2020; Zhu et al., 2020).

**Figure 1:**
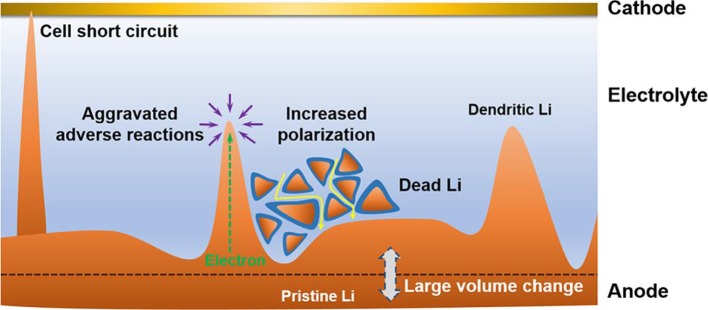
Scheme of the dilemma of Li metal anodes in rechargeable batteries. Reprinted with permission from Cheng et al. (2017) Copyright (2017) American Chemical Society.

Li metal has the highest theoretical capacity (3,860 mAh g^−1^ or 2,061 mAh cm^−3^) and lowest electrochemical potential (−3.04 V vs. the standard hydrogen electrode), and consequently it has been considered as an ideal anode for the rechargeable batteries such as Li-S and Li-O_2_ batteries (Li et al., 2019; Wu et al., 2019). Unfortunately, Li tends to precipitate as dendrites when it is used as an anode material during charging. The formed dendrites would penetrate the separator, causing a short circuit. Moreover, the isolated Li will be produced upon discharging, thus inevitably lowering the cycling performance of Li metal anode. The severe problems due to the formation of Li dendrites have been reviewed in detail by Zhang et al. (Cheng et al., 2017), and they are illustrated in Figure 1, mainly including (1) cell short circuit, (2) aggravation of adverse reactions, (3) evolution of dead Li from the dendrites, (4) increased polarization, and (5) large volume change.

To cope with the above challenges, many approaches have been proposed to make Li metal anode viable in secondary batteries, including the electrolyte additives (Shiga et al., 2017), solid-state electrolytes (Kato et al., 2016; Ates et al., 2019), artificial solid electrolyte interphase (SEI) (Kozen et al., 2015), structured anode (Matsuda et al., 2017), and so on. On the other hand, some important theories such as the formation mechanism of SEI on Li metal anode, and the nucleating and growth of Li dendrites have also been proposed (Peled et al., 1997; Ely and Garcia, 2013; Xu et al., 2014). These investigations have greatly deepened our understanding on the subject of Li metal anode, and contributed much to the Li metal anode technology. Furthermore, they strength our confidence in the upcoming era of rechargeable batteries with ultra-high energy density.

However, on the whole, Li metal anode was in the infancy of LMB research. LMBs were firstly invented in 1976 as non-rechargeable batteries (Satter, 1976), but the safety problem caused by dendrite formation as well as the subsequent rise of LIBs make the research of LMBs become sluggish. The primary technical problems impeding the practical application of Li metal anode include the high reactivity, ample dedrite formation, huge volume fluctuations, poor cycling performance, and the safety issues (Cheng et al., 2017). Recently, with remarkable advances in scientific technologies, an increasing number of cutting-edge electron devices come into our daily life. Therefore, the demand for the rechargeable high-energy batteries becomes more and more pressing. Naturally, LMBs again attract considerable interest of scientists due to their inherent outstanding advantage of high energy density.

At present some researchers have reviewed the strategies to suppress the growth of Li dendrites in lots of papers, but they are mainly concentrated on some strategies instead of overall views (Yang and Li, 2018; Ko and Yoon, 2019; Zhao et al., 2020), while comprehensive understanding of various strategies is necessary to develop novel methods to solve the dilemmas of Li metal anode described above. In this review, we summarized various strategies to deal with the problems of Li metal anode related to its safety and cycling performance deterioration. The nature of each strategy was also discussed. Prior to the introduction of these strategies, some important theories and mechanisms related to Li metal anode have been elucidated to better understand these strategies. It is reasonably believed that with advanced characterization technique and nanotechnology, the problems hindering LMBs' application will be completely solved under the joint efforts of scientists all over the world, and the era of LMBs will come soon.

## Theories Related to Li Metal Anode

### Li Dendrite Formation and Growth

There are three main types of morphologies of Li dendrites (mossy, dendritic, and granular ones), but the different dendrites have a similar mechanism on their formation and growth (Osaka et al., 1997; Yamaki et al., 1998). During Li deposition process, the initially presented nucleation sites play a key role in deciding the subsequent precipitation nature/behavior of Li^+^ ions and the nucleation process will happen during each cycle under different conditions. Li dendrite growth is self-enhanced, and one representative theory explaining this phenomenon was proposed by Ding et al. (2013). Their investigation showed that protrusions with big curvature can induce a quite higher electric field at their tips, which are prone to attract more Li^+^ ions for precipitation, promoting further growth of the protrusions and evolving into dendrites finally. Another important discovery was presented by Xu and his coworkers. Their researches indicated that protrusions with hemispherical tips can perform three dimensional (3D) diffusion of Li^+^ ions, rather than one direction diffusion in flat surfaces, thereby accelerating Li precipitation on the tips (Xu et al., 2014).

### SEI

**Figure 2:**
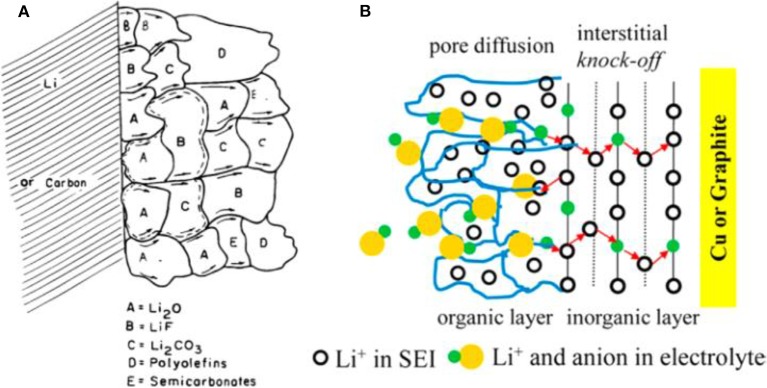
Scheme of SEI structure. **(A)** Mosaic structure in the top view. Reprinted with permission from Peled et al. (1997). Copyright (1997) The Electrochemical Society. **(B)** Dual-layer structure in the cross-section view. Reprinted with permission from Shi et al. (2012). Copyright (2012) American Chemical Society.

SEI was firstly discovered by Peled (1979), and it was formed on Li metal anode during the early charging/discharging cycles by decomposition reaction at the interface between the organic and Li (Peled, 1979; Collins et al., 2015). SEI is indispensable for a Li metal anode to operate normally. An ideal SEI film should be of excellent ionic conductivity and electronic insulation, as well as of a robust mechanical performance to adjust the irregular volume variation and a good stability upon the long-term cycling (Cheng et al., 2017). The structure and composition of SEI have been clarified by several models. The mosaic model is commonly accepted and its scheme is displayed in Figure 2A (Peled et al., 1997). Due to the highly reactive nature, Li electrode is usually covered with an inorganic layer including the components of Li_2_O, LiOH, and Li_2_CO_3_, which is formed by reaction with O_2_, H_2_O, and CO_2_ in air. After exposure to the electrolyte, an insoluble multiphase SEI film is formed simultaneously on the Li surface by reductive decomposition reactions. The obtained SEI with a mosaic morphology can make the Li^+^ ions migrate through it promptly. Another famous model is called dual-layer one which can explain the double layer structure of SEI [Figure 2B; Shi et al., 2012]. According to this model, the inorganic layer is adjacent to Li metal, which is often composed of Li_2_O_3_, LiF, LiOH, and Li_2_CO_3_, and the outside organic layer is made of ROCO_2_Li, ROLi, and RCOO_2_Li (where R is an organic group related to the solvent).

## Strategies to Improve the Performance of Li Metal Anode

The most severe problem of Li metal anode is Li dendrite growth on its surface, which not only causes catastrophic safety hazards, but also greatly deteriorates the performance of Li metal anode. Therefore, suppression of Li dendrite growth is prerequisite for each strategy. Nevertheless, some methods are effective to suppress Li dendrite formation, but they can also induce disadvantageous influence on the property of LMB at same time. Therefore, an optimal strategy should maximize the performance of Li metal anode while it can effectively suppress the formation of Li dendrites.

### Constructing SEI

#### Electrolyte Additive

**Figure 3:**
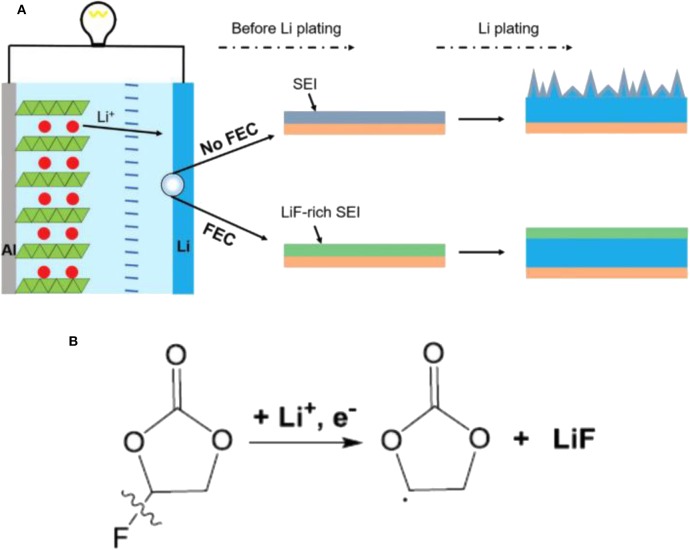
**(A)** Schematic illustration of the effect of the FEC additive on a Li metal anode. The electrolyte is 1.0 M LiPF_6_ in EC/DEC (1:1 by volume) with and without FEC additive. **(B)** Proposed possible mechanisms for the decomposition of FEC to produce LiF. Reprinted with permission Zhang X. et al. (2017). Copyright (2017) WILEY-VCH.

**Figure 4:**
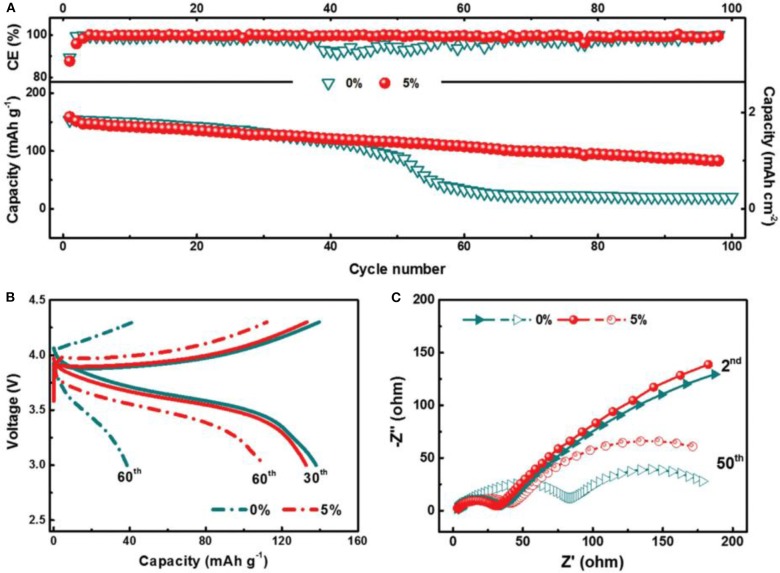
Electrochemical performance of Li|NMC cells with 0 and 5% FEC additive. **(A)** Cycling performance and **(B)** voltage profiles of the cells at 1.0 C, 3.0–4.3 V. **(C)** EIS of Li|NMC cells after 2 and 50 cycles. Reprinted with permission Zhang X. et al. (2017). Copyright (2017) WILEY-VCH.

It is well-known that SEI plays an important role in LMBs and strongly affects the surface chemistry of Li metal anodes (Zhao and Zhang, 2019). Appropriate Li salts in electrolytes can react with Li metal anode to form a thin, compact and uniform SEI layer, thus suppressing Li dendrite growth. However, there are only a few Li salts available for Li metal anode such as LiClO_4_, LiBr, and LiAsF_6_ (Zaban and Aurbach, 1995). Compared with Li salts, electrolyte additives have attracted more and more interest recently (Wang et al., 2019). They can ameliorate the physical-chemical properties of the SEI, and furthermore they can make the current distribute more evenly upon Li precipitation (Kim et al., 2013). Moreover, electrolyte additives tend to be very efficient, and sometimes even at ppm levels, they can considerably change the deposition morphology and cycling efficiency. There are mainly two categories of additives to be employed: inorganic and organic additives. Generally, inorganic additives are more commonly used, including HF, LiNO_3_, lithium polysulfide, and so on (Zhao et al., 2016; Yang and Li, 2018). The SEI film assisted by inorganic additive is of high ionic conductivity and excellent mechanical strength. In contrast, organic additives often induce a more flexible SEI film due to their inherent ductility. Fluoroethylene carbonate (FEC), pyrrole, and cyclic carbonate are several typical organic additives (Heine et al., 2015). Among them, FEC has been considered as a promising additive because FEC/LiF can facilitate the formation of a protective SEI with excellent flexibility and high ionic conductivity, which could resist the volumetric fluctuations of Li metal anode. Figure 3 shows the effect of the FEC additive on Li metal anode (Zhang X. et al., 2017). After adding FEC, the coulombic efficiency, impedance and surface morphology had been improved greatly. In the investigation, the formed SEI film with and without FEC additive had been studied in detail. The results showed the FEC additive can induce a LiF-rich SEI, which could effectively suppress the formation of Li dendrites, and further study indicated that LiF in the SEI comes from decomposition of FEC. As shown in Figure 4, the coulombic efficiency and capacity retention become more stable and the impedance change is relatively small from the 50th cycle using FEC additive.

#### Chemical and Physical Pretreatment

**Figure 5:**
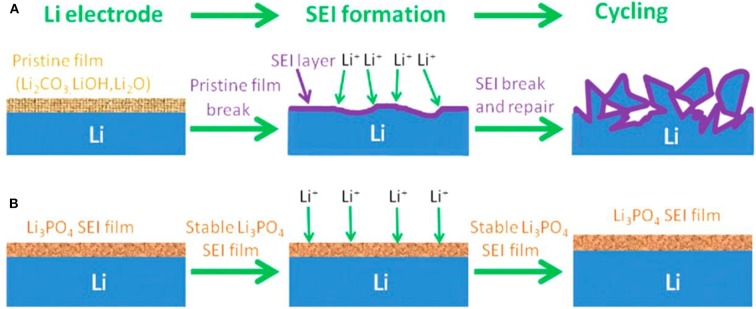
Scheme of Li metal anode with different SEI films. **(A)** General Li metal and **(B)** Li_3_PO_4_-modified Li metal anodes during SEI formation and cycling. Reprinted with permission from Li et al. (2016). Copyright (2016) WILEY-VCH.

The above described SEI films are very complicated, and their *in-situ* formation processes are difficult to control (Gauthier et al., 2015). Therefore, lots of methods have been developed to prepare a robust artificial film before cell operation and they have been proved to be efficient approaches to improve SEI layers. The artificial SEI layers can be constructed by chemical, and physical approaches. The chemical approaches mainly include the gas and liquid processing. The investigations performed by Koch et al. indicated that the Li_3_N passivation layer can be formed on Li surface by reacting Li with N_2_ at room temperature, and it is thought to have excellent stability against Li, thus well protecting Li metal (Koch et al., 2015). Besides the gas processing, liquid processing is another powerful technique to coat a defensive film on the surface of Li metal. Li et al. prepared a Li_3_PO_4_ uniformly distributed artificial SEI layer by *in-situ* reaction of polyphosphoric acid with Li metal and its native film (Li et al., 2016). As shown in Figure 5, the Li_3_PO_4_ layer can facilitate the transportation of Li^+^ ions between the electrolyte and Li metal interface, and furthermore it can induce a smooth surface and high Young's modulus that is sufficient enough to suppress the growth of Li dendrites by mechanical resistance. Other successful examples by liquid processing were the preparations of Cu_3_N layer, LiF layer, and so on (Liu Y. et al., 2017; Lang et al., 2019). The chemical approach can deliberately regulate the SEI components but limited to several ones. With the development of chemistry science, it will become a more effective method to selectively adjust the SEI film.

Physical approach can deposit inorganic materials (including carbon, Al_2_O_3_, LiF, Li_3_PO_4_, and so on) and organ polymers. Carbon is one of the commonly used inorganic materials for artificial SEI film. Zhang et al. have successfully coated a-CNx layer on the Li metal anode by magnetron sputtering technique (Zhang Y. et al., 2016). The a-CNx layer possesses such good flexibility and high mechanical strength that the stable and robust SEI film can effectively inhibit the Li dendrites growth. Al_2_O_3_ film has been often fabricated by spin-coating and atomic layer deposition (Jing et al., 2015; Chen et al., 2017). The Al_2_O_3_ films prepared by different physical approaches possess different structures and morphologies, but all of them can effectively suppress Li dendrite growth. Organic polymers have an excellent flexibility and they are likely to remit the volume change upon Li cycling. However, generally a polymer has poor ionic conductivity, naturally the construction of well-organized channels for Li^+^ ions in the polymer is highly desired. Zhu and his coworkers prepared a modified poly(dimethylsiloxane) (PDMS) layer using spin-coating method, and then the layer was treated by HF acid, intentionally constructing nanopores as the pathways for transporting Li^+^ ions in the PDMS layer (Zhu et al., 2017). By adjusting the etching time, the excellent PDMS lay with a 500 nm thickness and the pore sizes of 40–100 nm was obtained. The as-prepared PDMS layer possesses excellent ionic conductivity, good mechanical, and chemical stability against Li metal anode. Moreover, the PDMS SEI layer induces a stable cycling for 200 cycles with a high coulombic efficiency of 94.5% at a current density of 0.5 mA cm^−2^. The physical approach is a simple and effective strategy to protect Li metal anode, and the layer can also be considered as one kind of solid electrolyte.

### Engineering of Solid-State Electrolytes

The liquid electrolytes commonly used in LIBs are inappropriate for LMBs because they can raise severe safety issues such as leakage, poor chemical stability, and flammability. On the contrary, utilization of solid-state electrolyte (SSE) can probably settle these safety issues (Xu et al., 2014; Wu et al., 2020). Moreover, the SSEs possess high modulus and thus can effectively suppress Li dendrite growth. There are three kinds of SSEs to be developed, including solid inorganic electrolyte (SIE), solid polymer electrolyte (SPE), and inorganic/polymer hybrid electrolyte. At present, the researches on inorganic electrolytes become sluggish due to their brittleness and instability against Li and in air. Similar to inorganic electrolytes, polymer electrolytes also have to face the issues such as low ionic conductivity and poor stability against Li. Consequently, the composite electrolytes have been considered as promising candidate electrolytes for LMBs.

#### Inorganic/Polymer Hybrid Electrolyte

Inorganic/polymer hybrid electrolyte can hopefully fulfill the demand of SSE for LMBs in that it combines the advantages of each component and overcomes its deficiencies by synergetic effect among them (Sun et al., 2017; Zhang et al., 2020). By compositing, the composite electrolyte is expected to own good mechanical flexibility and high ionic conductivity. The mechanical flexibility is much related to the polymer amount while the ionic conductivity is greatly influenced by the compositing way of the inorganic and polymer components. Because the ionic conductivity plays a key role in the composite electrolyte, we will focus the following means to facilitate ion transport in the composite in this section.

**Figure 6:**
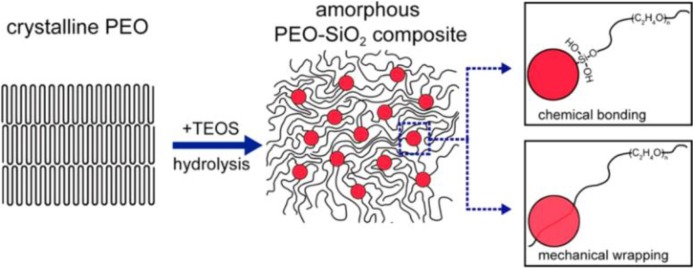
Scheme of *in situ* hydrolysis and interaction mechanisms among PEO chains and MUSiO_2_. Reprinted with permission from Lin et al. (2016a). Copyright (2016) American Chemical Society.

1. Synthesis method, including mechanical mixing and *in-situ* synthesis. The mechanical mixing method is most extensively used to synthesize the composite electrolyte due to its simplicity and conveniency. Nevertheless, the as-obtained composite electrolyte tends to have an uneven distribution of the inorganic and polymer electrolytes, resulting in the aggregation of particles, which will inevitably lower the ionic conductivity. In contrast, *in-situ* synthesis can render the uniform distribution of the two components. Lin et al. prepared PEO-SiO_2_ composite electrolyte by *in-situ* hydrolyzing TEOS in crystal PEO polymer (Lin et al., 2016a). As shown in Figure 6, monodispersed SiO_2_ nanospheres with a size of 12 nm were homogeneously incorporated in the PEO matrix, and they were strongly connected with PEO by chemical/mechanical interactions, thus significantly decreasing the crystallinity of PEO as well as promoting polymer segmental motion for ion transport. The thus-obtained composite electrolyte possesses an excellent conductivity (4.4 × 10^−5^ S cm^−1^ at 30°C, 1.2 × 10^−3^ S cm^−1^ at 60°C).
2. Nanosized inorganic electrolytes. It is known that nanocrystallization can lead to high ionic conductivity because it can improve the diffusion routes for Li^+^ ions. In the composite electrolyte, polymer can act as a binder to connect the inorganic electrolyte while maintaining high mechanical flexibility. The investigation conducted by Zhang et al. indicated that the utilization of nanosized solid inorganic electrolyte can greatly increase its contact area with PEO, and thus it significantly enhances the ionic conductivity of the composite electrolyte (Zhang J. et al., 2016). Moreover, the tiny size of the inorganic electrolyte filler is helpful to retain the strong mechanical flexibility. The ionic conductivity of the nanosized filler is enhanced to be 2 orders of magnitude larger than that with the microsized one (10^−4^ S cm^−1^ at 30°C).
3. Adoption of one dimensional inorganic fillers. The traditional inorganic filler is nanoparticles, which are of zero dimensionality (D). Compared with 0D inorganic particles, 1D nanowire filler can induce larger contact area between the inorganic and polymer electrolytes, hence ameliorating the mechanical performance. In addition, 1D inorganic fiber fillers are very beneficial to form an ionic conduction networks in the composite electrolyte, significantly increasing the ionic conductivity of the electrolyte. Fu et al. synthesized a garnet-type Li_6.4_La_3_Zr_2_Al_0.2_O_12_ (LLZO) nanofiber by electrospinning way, and then added the fillers into the PEO matrix, resulting in a 3D inorganic/polymer network. In the electrolyte, the randomly distributed nanofibers together with the interconnected nanofibers constitute the 3D networks, which are the framework for transporting continuous Li^+^ ion (Fu et al., 2016). On the other hand, the researches on the mechanisms of ionic conductivity indicated that the LLZO phase is the main pathway for transporting Li^+^ ions (Zheng et al., 2016).

#### Electrode/Electrolyte Interface

Apart from the ionic conductivity, the interfacial resistance between electrode and electrolyte is another important issue in the solid-state electrolyte.

Recently the electrode tends to adopt nanosized active materials while the electrolytes are still in micron size. The intimate contact should be maintained between nanosized active materials and micron sized electrolytes to decrease the interfacial resistance and increase the stability. Some strategies have been proposed to decrease the interfacial impedance by coating lithiophilic materials, such as amorphous Si, ZnO, and Al_2_O_3_ with an atomic layer deposition technique (Kozen et al., 2015). The coating can tightly link Li metal and the electrolyte, thus drastically reducing the impedance. The intermediate layer can also be formed through the *in-situ* reaction between the electrolyte and Li metal, which has been proved to be a useful method. For example, Li et al. made LiZr_2_(PO_4_)_3_ with a rhombohedral structure react with Li metal anode at room temperature to form a Li^+^-conducting passivation layer (Li et al., 2016). The layer contains Li_3_P and Li_8_ZrO_6_, and they can wet LiZr_2_(PO_4_)_3_ and also are wet by Li, thus resulting in a small Li^+^ charge transfer resistance at the interface. Recently, introduction of a third phase is commonly used to decrease the interfacial resistance. For example, addition of little liquid electrolyte can effectively wet the solid-state electrolyte, and ameliorate the contact between the Li metal anode and electrolyte, considerably decreasing the interfacial resistance (Liu B. et al., 2017).

### Adoption Matrix for Li Metal Anode

As a hostless electrode, Li metal anode has vast volume fluctuations upon charging/discharging cycles, which would certainly lower the coulombic efficiency and decrease the mechanical stability. Therefore, adopting an appropriate matrix has been proposed to solve this problem. An efficient matrix could not only substantially regulate the volume change of Li anode during charging/discharging, but also overwhelm the growth and formation of Li dendrite by adjusting the Li plating/stripping behavior.

#### Lithiophilic Matrix

Based on the related theory of Li dendrite growth, the unevenly distribution of Li^+^ ions and current density on the current collector is the main reason for the growth of Li dendrites (Ding et al., 2013; Xu et al., 2014). Hence Li dendrites would be well-inhibited if the Li^+^ ions are uniformly distributed on the anode surface.

**Figure 7:**
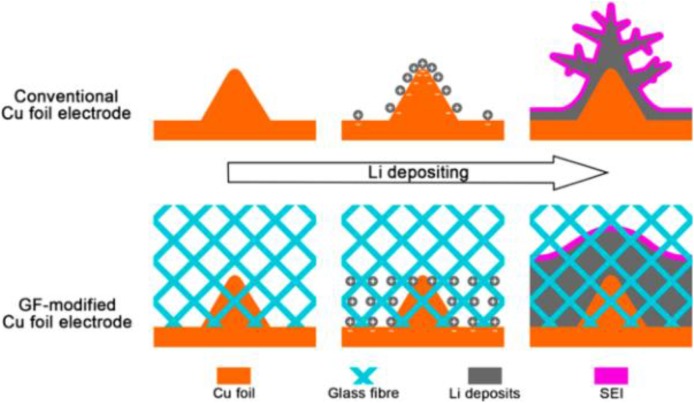
Scheme of Li deposition under the protection network of polar fiber membrane. The electrode of 2D Cu sheet always presents an uneven surface. The cloth of glass fibers (GFs) keeps large numbers of polar functional groups that can attract the spatial charges above the Li metal. The concentrated Li^+^ ions through the protuberances on the surface of Cu foil electrode are uniformly plated, consequently, hindering the Li dendrite formation on a working Li anode. Reprinted with permission from Cheng et al. (2016). Copyright (2016) WILEY-VCH.

Liang et al. utilized lithiophilic oxidized polyacrylonitrile nanofiber (PAN) as a matrix to modify Li metal anode and the investigation indicated that the nanofibers can maintain the stable cycling performance of Li metal anode with a high coulombic efficiency (~97.4%) for 120 cycles at the current density of 3 mA cm^−2^ and 1 mAh cm^−2^ (Liang et al., 2015). Metal-organic framework, N-doped graphene and N-doped PAN can also act as the host materials to enable stable Li charging/discharging with dendrite-free by chemical interactions (Liu W. et al., 2017; Matsuda et al., 2017; Zhang R. et al., 2017). Linking glass fibers (GFs) with rich polar functional groups on the anode surface can easily and uniformly distribute Li^+^ ions (Cheng et al., 2016). As displayed in Figure 7, the polar functional groups in GFs made the binding energy of Li/glass fiber 1.0 eV higher than that of Li/Cu foil. The higher binding energy can make GFs adsorb more Li^+^ ions while protuberances of Cu foil or the previously grown Li dendrites will hardly incur Li^+^ ions due to weak electrostatic interactions. By this method, the accumulation of Li^+^ ions can be prevented and Li dendrite growth can be effectively suppressed. At different densities (0.5, 1.0, 2.0, 5.0, and 10.0 mA cm^−2^), cells with GF layers exhibited enhanced coulombic efficiencies (98, 97, 96, 93, and 91%, respectively). Using graphene oxide (GO) as a porous matrix for Li metal, Lin et al. prepared Li-reduced oxide (rGO) by a facile “spark” technique (Lin et al., 2016b). When the GO sheets partially come into contact with molten Li, a spark reaction will happen until the whole GO sheets are filled with molten Li. The spark reaction can convert non-conductive GO into conductive rGO and render molten Li to enter the GO matrix. The Li-rGO anode can display a dendrite-free morphology upon Li plating and show a good rate performance (60 mAh g^−1^) at 10 C using lithium cobalt oxide cathode.

#### Conductive Matrix

A Li metal anode with a conductive matrix possesses excellent electrical conductivity and unique surface chemistry, which can obstruct and recycle the dead Li. Adoption of conductive matrix for Li anode has been demonstrated to be a successful approach to inhibit Li dendrite formation (Cheng et al., 2015). Ryou et al. utilized a microneedle surface to cover an extensive surface area of Li electrode (Ryou et al., 2015). Besides suppression of Li dendrite, this strategy can improve the rate capability and cyclability by reducing interfacial resistance (Park et al., 2016). However, the Li metal anode prepared by this approach has limited lithiation capacity and short lifespan. Recently, the 3D conductive matrix has been attempted to modify Li metal anode due to their high surface area and high electric field intensity. The matrix can attract Li^+^ ions to plate in it, instead of on the tip of the formed Li dendrites, consequently suppressing the Li dendrite growth. This assumption was verified by preparing Li_7_B_6_ with 3D structure (Cheng et al., 2014). The Li_7_B_6_ anode achieved a dendrite-retarded morphology at a high current density of 10 mA cm^−2^, while the normal Li plate anode rendered severe dendrite formation. Besides Li_7_B_6_, several structured Li metal anodes have been also developed, such as carbon nanotubes, graphene materials, carbon nanofibers, and Cu nanowires. The matrix plays a pivotal role for Li metal anode in that it can not only suppress Li dendrite growth, but also effectively relieve the large volume change during Li plating/stripping. However, the ratio of the matrix in the composite Li metal anode needs to optimize to ensure the high volume/mass energy density of the composite anode.

For better grasping the essential of the strategies in this review, we summary the main purpose and characteristic for each strategy/method, which are listed in Table 1. Based on these strategies, it is expected that novel approaches/methods would be developed to better solve the problems hindering the application of Li metal anode.

**Table 1:** Purpose and characteristic for each strategy/method.

**Strategy/method**	**Purpose, nature and characteristic for each strategy**	**References**
Constructing SEI	To prolong the cycle life of Li anode	(Zhao and Zhang, 2019)
Electrolyte additive	To form a SEI film with high conductivity and compactness	(Wang et al., 2019)
Chemical pretreatment	To intentionally regulate SEI film	(Lang et al., 2019)
Physical pretreatment	To induce a robust and stable SEI film	(Zhang Y. et al., 2019)
Engineering of solid-state electrolytes	To improve the safety of LMBs	(Wu et al., 2020)
Inorganic/polymer hybrid electrolyte	To achieve high ionic conductivity and mechanical flexibility	(Zhang et al., 2020)
Electrolyte/electrode interface	To reduce the interfacial resistance and instability	(Li et al., 2016)
Adoption matrix for Li anode	To minimize volume change using stable host	(Cheng et al., 2017)
Lithiophilic matrix	To induce a uniform distribution of Li^+^ ions on the surface of Li anode	(Zhang R. et al., 2017)
Conductive matrix	To inhibit dead Li and recycle dead Li	(Park et al., 2016)

## Conclusion and Outlook

To make Li metal anode to be a viable technology, many strategies have been proposed to overcome the dilemmas of Li metal anode, including the growth of Li dendrites, volume fluctuation, low mechanical strength, and high interfacial resistance between electrolyte and electrode. In this review, we outlined the main strategies to improve Li metal anode according to the classifications of constructing SEI, engineering of solid-state electrolyte and adopting matrix for Li metal anode. For better understanding, we illustrated the preparation, characteristic and mechanism of each strategy in detail by exemplification. Although great progress has been made in theoretical and experimental investigations, Li metal anode is in the early stage of LMBs development. We think that the following researches on Li metal anode should be further strengthened.

1. More detailed and deeper theories on Li anode should be developed to guide the design of SEI, solid-state electrolyte and structured anode. For example, several models have been proposed to explain Li nucleation and growth, but they are limited to some specific parameters. A more general mechanism should be need to understand the whole process for Li dendrite growth. For another example, previous theories held that the Li^+^ ions transport through the interface of ceramic/polymer in the inorganic/polymer electrolyte while later investigations demonstrated that the ceramic phase is the transport channel for Li ion. This discovery rendered a revolution in designation of the composite electrolyte.
2. A joint strategy is required to solve the problems of Li metal anode. Although each strategy has its unique advantage to deal with a dilemma of Li metal anode, it also has its own fatal weakness. For example, solid polymer electrolyte has enough mechanical strength to suppress the Li dendrite, but its ionic conductivity is very low. The joint strategy combining various methods can fully takes advantages of each method, and the synergetic effect among them could ultimately make Li metal anode a viable technology.

With the development of science and technology, the advanced electronic devices are successively emerging and they urgently need high energy density batteries as their power sources. In addition, the rise of nanotechnology and advanced characterization technique render the golden age for LMBs researches. The problems hindering the application of Li metal anode will be perfectly solved, and the era of LMBs will come true in the near future.

## Author Contributions

All authors wrote the manuscript. ZH and YZ supervised the manuscript.

## Conflict of Interest

The authors declare that the research was conducted in the absence of any commercial or financial relationships that could be construed as a potential conflict of interest. The handling editor declared a past co-authorship with one of the authors YZ.
